# The “true” acetabular anteversion angle (AV angle): 2D CT versus 3D model

**DOI:** 10.1007/s11548-022-02717-w

**Published:** 2022-07-27

**Authors:** Kira A. Barlow, Zdzislaw Krol, Pawel Skadlubowicz, Chao Dong, Vanja Zivkovic, Andreas H. Krieg

**Affiliations:** 1 Pediatric Orthopedic Department, University Children’s Hospital Basel (UKBB), Spitalstrasse 33, CH-4056 Basel, Switzerland; 2 Institute of Computer Science, Department of Biomedical Computer Systems, Faculty of Computer and Materials Science, University of Silesia, Sosnowiec, Poland

**Keywords:** Acetabular anteversion, 3D-CT, Pediatric orthopaedics, Pelvic osteotomy, Acetabular anatomy, Radiology, Preoperative planning

## Abstract

**Introduction:**

Different factors can lead to inconsistencies in measurement for the acetabular version using 2D axial CT-cuts. We have defined a “true” anteversion angle (AV angle) in the physiological position of the pelvis in 3D with the largest European population measured to our knowledge.

**Material and methods:**

We analyzed 258 hemipelvises and created 3D models. We compared the results of our AV angle 3D method with the cross-sectional cuts of the same acetabula. We included factors like side, sex, body mass index, and patient positioning.

**Results:**

Overall, the mean (SD) AV angle was 16.1 (5.9)° as measured with the 3D method and 22.0 (6.0)° as measured with the 2D method (*p* < 0.0001). Measured with both the 3D and the 2D method, the AV angle was significantly larger in female than in male individuals (*p* < 0.0001).

In the 2D method, the AV angle estimation was influenced by the pelvic tilt.

**Conclusion:**

We propose a more accurate method for the measurement of the AV angle of the acetabulum in a 3D model that is not influenced by patient positioning or pelvic tilt. We provide a computational model that will facilitate operative decisions and improve preoperative planning. We confirm that 3D measurement should be the gold standard in measuring the acetabular anteversion.

## Introduction

Murray et al. [15] have defined the acetabular angles as “anatomical anteversion” (AA) and “anatomical inclination” (AI). AA is defined as the angle between the transverse axis and the acetabular axis in the transversal plane, whereas AI is defined as the angle between the acetabular axis and the longitudinal axis, which is often referred to as the “anteversion of the acetabulum”. AA and AI can only be evaluated in a 2D plane.

Various methods have been described to assess the acetabular anteversion in 2D CT scans [20]. In 2009, 3D CT scans have been described to be more accurate for the analysis of the acetabula [5]. However, those measurements are dependent on the position of the pelvis in the CT scanner [5]. The bodyweight and constitution of patients also influences the position of the pelvis and the 2D measurements. Therefore, a more accurate analysis of the true positioning of the pelvis with 3D reconstruction is needed. Tönnis et al. [22] tried to avoid the effect of pelvic tilt: they defined a plane between the superior anterior iliac spines and the symphyses and measured the angles in correlation to that.

Retroversion of the acetabulum leads to abnormal weight bearing on the acetabular surface, and therefore to instability and mechanical impingement. This can lead to cartilage lesions and early osteoarthritis [7]. Knowledge of the normal orientation of the acetabulum is essential for the diagnosis of the type and severity of developmental dysplasia of the hip (DDH), as well as for the preoperative planning. Accurate estimation of the normal contact surface orientation permits correct realignment of the osteotomized acetabulum.

We have defined a “true” anteversion angle (AV angle) in the physiological position of the pelvis in 3D reconstructions. The aim of our study was to measure this “true” AV angle in the healthy population and show the true anatomic situation. Our hypothesis was that the 3D measurement is more accurate than the 2D measurement.

## Materials and methods

### Patient data

This study was approved by the local ethics committee (Ethikkommission Nordwest- und Zentralschweiz EKNZ 343/08). We studied 258 hemipelvises (acetabula) that had been CT-scanned for abdominal evaluation (129 subjects). The CT scanners used in this study were GE LightSpeed 16 (GE Healthcare, Chicago, USA) and Siemens Sensation 16 (Siemens Healthineers, Munich, Germany). The voxel sizes in the CT data pool ranged from 0.66 to 0.75 mm (in-plane resolution) and from 1.0 to 1.5 mm (slice thickness). Patients’ baseline characteristics are shown in Table 1.

**Table 1:** Demographic data (n = 129)

	Overall	Male (*n* = 68)	Female (*n* = 61)
	Mean (SD); range	Mean (SD); range	Mean (SD); range
Weight (kg)	74.7 (14.9); 48–120	81.1 (14.2); 48–120	67.5 (12.3); 48–96
Height (cm)	169.9 (9.9); 148–198	175.5 (8.5); 162–198	163.4 (7.2); 148–178
Age (years)	62.7 (15.5); 20–88	62.0 (16.2); 20–88	63.5 (14.7); 26–86
BMI	25.9 (4.8); 16.8–41.0	26.3 (4.3); 16.8–35.6	25.5 (5.5); 17.2–41.0

Inclusion criteria were the following: (a) age 18 to 85 years; (b) available bilateral 3D CT images of the pelvis, taken at a single hospital; (c) availability of written informed consent; (d) no history of hip-related surgery. Exclusion criteria were the following: (a) history of hip related surgeries; (b) history of hip-related disease including infection, trauma, and neoplasm; (c) incomplete scans. The patients were retrospectively included in the study.

### Analysis of the CT data: 2D

The AV angle was first measured in 2D using the cross-sectional computation method by Stem et al. [20]. The 2D AV angle has been defined as an angle between the transischial line across the ischial tuberosities on the axial CT image and a second line drawn across the margins of the bony acetabulum. The measurements were taken in the axial plane, which is the closest to both centers of the femoral heads.

The alternative 2D computation method is described by Tallroth et al. [21]. The main difference is the reference line drawn perpendicular to a line defined by the centers of the femoral heads.

### Analysis of the CT data: 3D

**Fig. 1:**
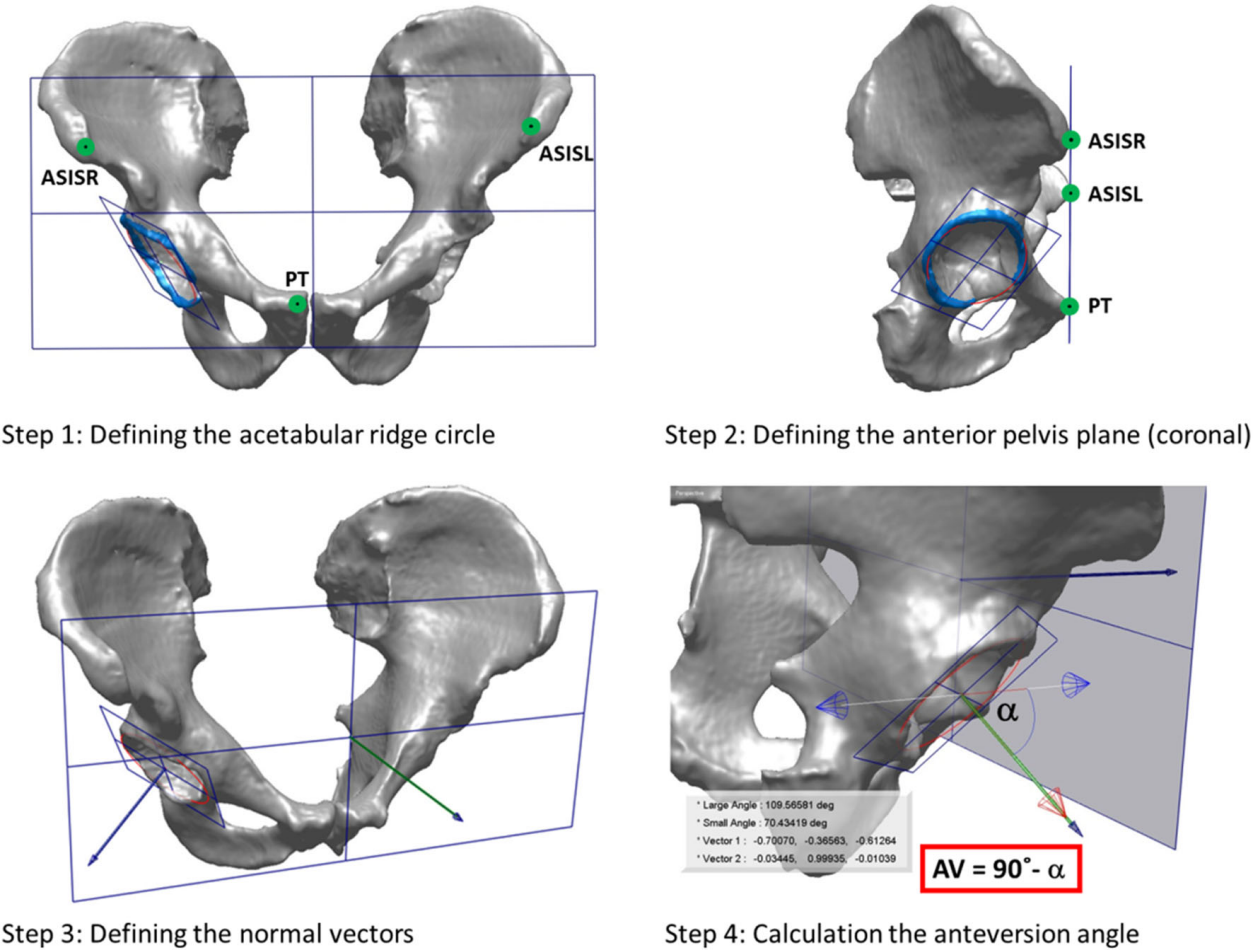
Steps of the 3D measurement: 1 – defining the acetabular ridge circle (red) and the coplanar acetabular ridge plane; 2 – defining the anterior pelvis plane (coronal) through three anatomical landmarks: Anterior Superior Iliac Spine, left (ASISL) and right (ASISR), and the most forward protruding point of the pubic tubercles (PT); 3 – defining the normal vectors for the previously defined planes; 4–calculation of the AV angle as AV = 90° − *α*, with *α* being the angle between both normal vectors. The AV-angle in the 3D model, based on the above-described steps, can then be assessed by estimation of the angle between the normal vector of the acetabular plane and the normal vector of the anterior coronal plane (*α*), it is thus the complementary angle to *α*, i.e., AV = 90° − *α*. dataset

The segmentation process consists of the identification of the left and right ilium osseous structures in the CT data by using the segmentation software. The segmented bony structures (see Fig. 1) in each of the evaluated CT datasets were saved as standard triangle language (STL) datasets (3D triangle meshes). The STL format was created in the context of the stereolithography, which is a form of 3D printing technology, and has been established as the standard data format in the computer-aided design (CAD) software [9].

3D pelvic models were created for both acetabula (258 hemipelvises). On the 3D models, the true AV angle was calculated using RapidForm—a 3D scan data processing package offered by INUS Technology, Inc. (Seoul, South Korea) as well as our own developed *Sevismo* and *Landmarks* software.

The definition of the required planes, lines, vectors and landmarks in the generated 3D virtual surface model of the pelvis was based on the analogous anatomical definitions from the literature [14]. The anatomical landmarks (see Fig. 1) were manually labeled by an orthopedist for each case. Two other orthopedists reviewed the initial labeling. If there was any disagreement, the three orthopedists consented on one optimal landmark placement which was used for further calculations. By manual labeling, possible inaccuracies, such as osteophytes, were eliminated. The crucial step in the evaluation of the AV-angle in the 3D pelvic model was the delineation of the anterior ridge of the acetabulum as shown in blue color in Fig. 1 (*upper part)*. The required acetabular rim can be automatically identified by generation of the bone surface curvature plot followed by the selection of the most curved area on the acetabular margins. The delineated acetabular ridge triangle mesh consists of on average 2500 vertices (points). A mathematically optimal circle fitting procedure (with removing outliers) was applied to these points (see red circle in Fig. 1), defining the acetabular plane that was used, together with the coronal plane, as the basis for the estimation of the AV-angle in the 3D model. All the consecutive steps of the 3D computation method for the acetabular AV angle are shown in Fig. 1.

### Statistical analysis

Quantitative data are presented as mean ± standard deviation (SD). The statistical analysis software packages used are Microsoft Excel (Richmond, VA, USA), GraphPad Prism (Version 8.0, San Diego, CA, USA) and SPSS 23.0 (IBM Corporation, Armonk, NY, USA). The means were compared by a *t* test for independent samples. The correlation between the difference of 2D and 3D methods and the possible impact factors was analyzed by single and multiple linear regression. A *p*-value less than 0.05 was considered statistically significant.

## Results

**Fig. 2:**
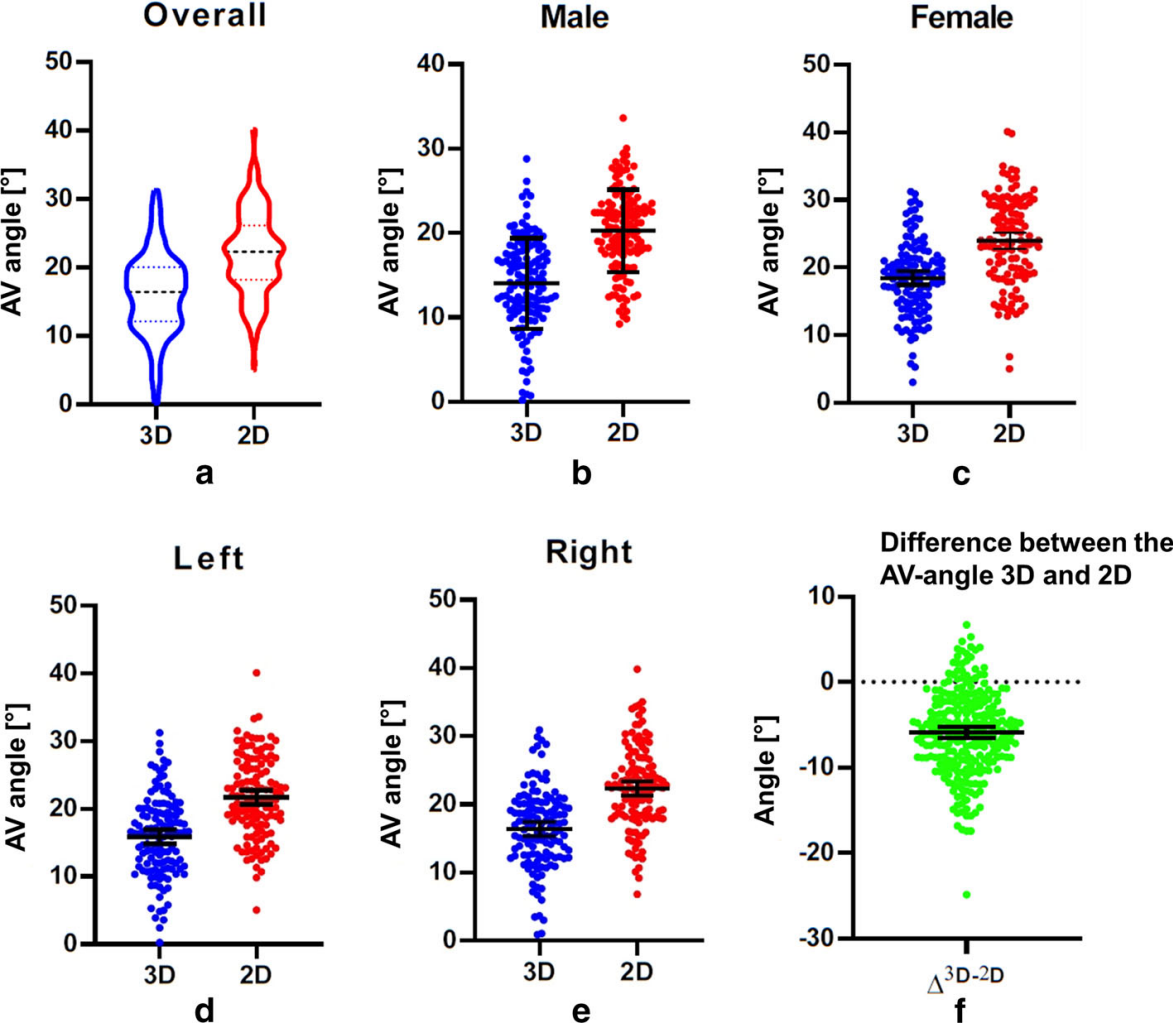
**a** Overall comparison of the AV angles as measured with the 3D and 2D method. The tendency of both methods is similar, with a mean (SD) 16.1 (5.9) in the 3D group and 22.0 (6.0) in the 2D group. **b**, **c** Comparison of the AV angles in the males and females as measured with the 3D and 2D method. **d**, **e** Comparison of the AV angles on the left and right as measured with the 3D and 2D method. **f** The difference between the AV angles as measured with the 3D and 2D method. The mean (SD) distance is − 5.88 (4.92) degrees, meaning that the angle as measured with the traditional 2D method is 5.88 degree larger than the angle as measured with the 3D method

The mean AV-angle measured by the 3D method (AV^3D^) was 16.1° (SD = 5.9°), and 22.0° (SD = 6.0°) with the 2D method (AV^2D^). The mean (SD) difference between AV^3D^ and AV^2D^ was 5.8 (4.9)° (p < 0.0001), which means that the AV^2D^ has an on average 5.8° larger bias (see Table 2 and Fig. 2a, f).

**Table 2:** Comparison between AV^3D^ and AV^2D^, overall patients, males and females, right and left subgroups (*Comparison between male and female, **Comparison between 3 and 2D, *** Comparison between left and right)

	Gender			BMI			
	Female (*n* = 61)	Male (*n* = 68)	*p*-value^**^	< 18.5 (*n* = 6)	18.5–25 (*n* = 54)	> 25 (*n* = 69)	*p*-value^***^
AV^3D^ left, m (SD)	18.15 (5.77)	13.77 (5.32)	< 0.001	17.53 (3.66)	15.96 (5.99)	15.61 (6.08)	0.6934
AV^2D^ left, m (SD)	23.39 (6.49)	20.15 (4.94)	< 0.001	23.34 (4.16)	21.12 (6.37)	21.97 (5.72)	0.5548
*p*-value^*^	< 0.0001	< 0.0001		0.0109	< 0.0001	< 0.0001

In the male subgroup, the mean (SD) AV^3D^ was 14.0 (5.4)° and the mean AV^2D^ was 20.3 (4.9)°. The mean (SD) difference between the AV^2D^ and AV^3D^ was 6.2 (4.5)° (*p* < 0.0001); when compared with the AV^3D^, the AV^2D^ had an on average 6.2° larger bias (see Table 2 and Fig. 2b).

In females, the mean (SD) AV^3D^ was 18.4 (5.6)° and the mean AV^2D^ was 23.9 (6.5)°. The mean (SD) difference between the two methods was 5.5 (5.4)° (*p* < 0.0001); when compared with the AV^3D^, the AV^2D^ had an on average 5.5° larger bias (see Table 2 and Fig. 2c).

In the same-side comparison, on the right side, the AV^2D^ had a mean (SD) 5.9 (5.2)° larger bias than the AV^3D^ (*p* < 0.0001); on the left side, the AV^2D^ had a mean (SD) 5.8 (4.7)° larger bias than the AV^3D^ (*p* < 0.0001) (see Table 2 and Fig. 2d-e).

Patients with BMI < 18.5 showed larger AV angle estimation, both in 3D and 2D, than patients with a normal BMI and patients with a BMI > 25. However, these results were not statistically significant (*p* > 0.05; see Tables 3 and 4).

**Table 3:** Comparison on the left side between 3 and 2D in Gender and BMI (*Comparison between 3 and 2D method, **Comparison between male and female subgroups, *** Comparison between different BMI subgroups)

	Gender			BMI			
	Female (*n* = 61)	Male (*n* = 68)	*p*-value^**^	< 18.5 (*n* = 6)	18.5–25 (*n* = 54)	> 25 (*n* = 69)	*p*-value^***^
AV^3D^ left, m (SD)	18.15 (5.77)	13.77 (5.32)	< 0.001	17.53 (3.66)	15.96 (5.99)	15.61 (6.08)	0.6934
AV^2D^ left, m (SD)	23.39 (6.49)	20.15 (4.94)	< 0.001	23.34 (4.16)	21.12 (6.37)	21.97 (5.72)	0.5548
*p*-value^*^	< 0.0001	< 0.0001		0.0109	< 0.0001	< 0.0001

**Table 4:** Comparison on the right side between 3 and 2D in Gender and BMI (*Comparison between 3 and 2D method, **Comparison between male and female subgroups, *** Comparison between different BMI subgroups)

	Gender			BMI			
	Female (*n* = 61)	Male (*n* = 68)	*p*-value^**^	< 18.5 (*n* = 6)	18.5–25 (*n* = 54)	> 25 (*n* = 69)	*p*-value^***^
AV^3D^ right, m (SD)	18.70 (5.42)	14.31 (5.36)	< 0.001	18.08 (3.92)	16.69 (6.15)	16.00 (5.69)	0.5302
AV^2D^ right, m (SD)	24.48 (6.52)	20.62 (4.82)	< 0.001	24.88 (2.95)	21.62 (6.56)	22.63 (5.77)	0.2383
*p*-value^*^	< 0.0001	< 0.0001		0.0109	< 0.0001	< 0.0001	

Our study provides the highest number of evaluated patients in the so far published literature.

### AV-angle difference in the 3D and the 2D methods by position change of the patient

**Fig. 3:**
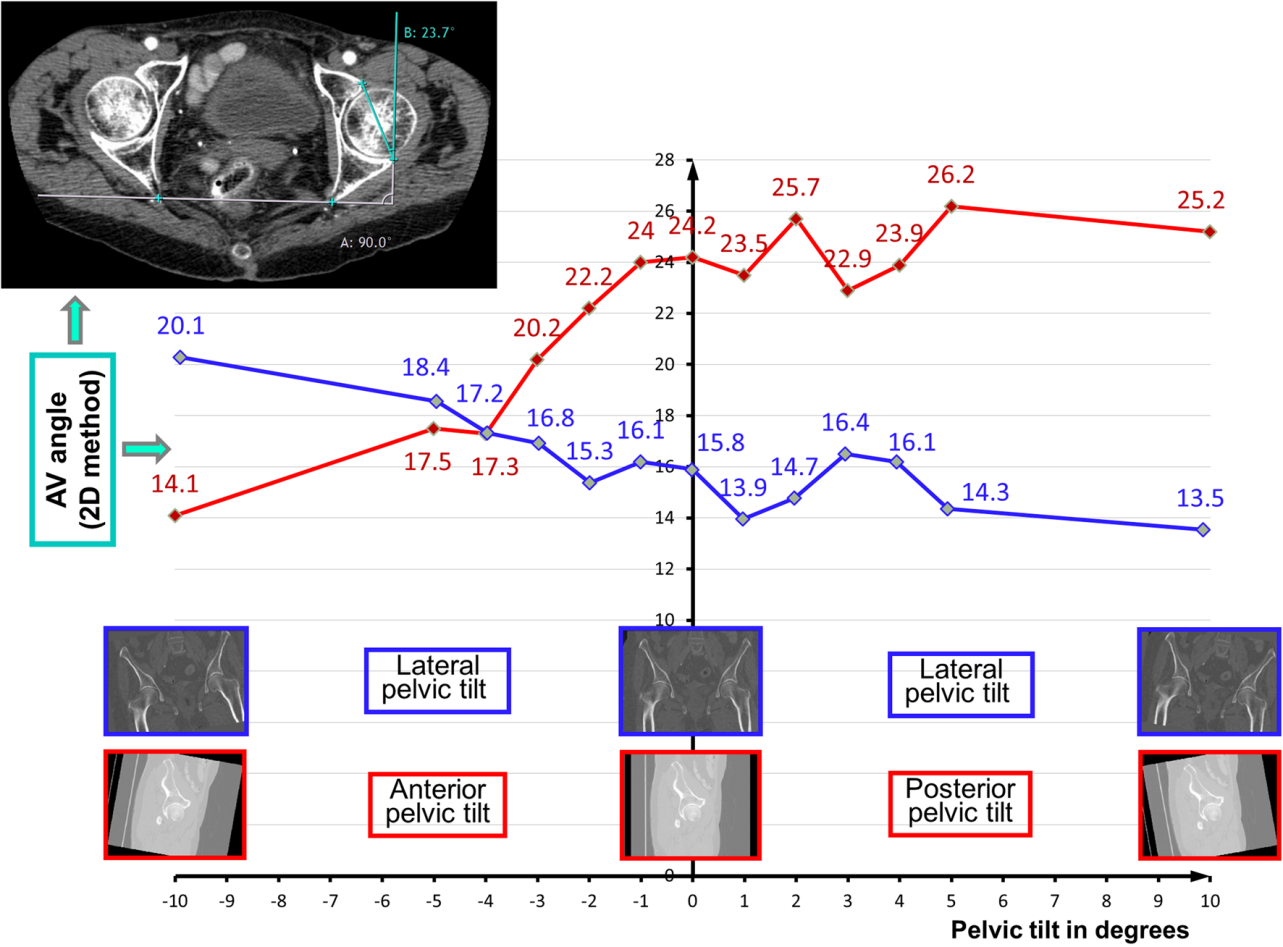
The 2D acetabular AV angle measurement in a single cross section of the correspondent CT dataset by the method of Stem et al. [20] used in our evaluation (the angle is measured between a line between the anterior and posterior acetabular ridge and a reference line drawn perpendicular to a line between the posterior pelvic margins at the level of the sciatic notch (see *upper left corner*)—is not rotation invariant. The simulations show the AV angle estimation differences by using of the 2D method due to variation of the anterior–posterior pelvic tilt (*red*) as well as the lateral pelvic tilt (*blue*)

The standard dorsal decubitus position of the patients’ pelvis during the CT data acquisition and the resulting pelvic tilt had a strong impact on the measurement of the AV angle, when measured in 2D. The following simulation reveals the influence of two rotation types on the 2D method: when we virtually changed the pelvic tilt from anterior to posterior (or vice versa; − 10° to 10°; see Fig. 3), the estimated AV angles showed a difference in a range from 14.1° when tilted anteriorly, to 25.2° when tilted posteriorly. The rotation of the pelvis around the vertical axis of the scanner table (lateral pelvic tilt) also had an impact on the estimation of the AV angle (see Fig. 3): the measured AV^2D^ was between 13.5° and 20°. The 3D method proved to be independent of the position of the pelvis on the scanner table during the CT acquisition.

### Correlation analysis of the AV-angle difference in the 3D and the 2D methods

To asses which rotation of pelvis induces the difference between AV^2D^ and AV^3D^, we used a single and multiple linear regression to analyze the rotations of the pelvis in the sagittal and coronal plane. The difference between the AV angles estimated by the 3D and 2D methods is denoted as Δ^3D−2D^.

**Fig. 4:**
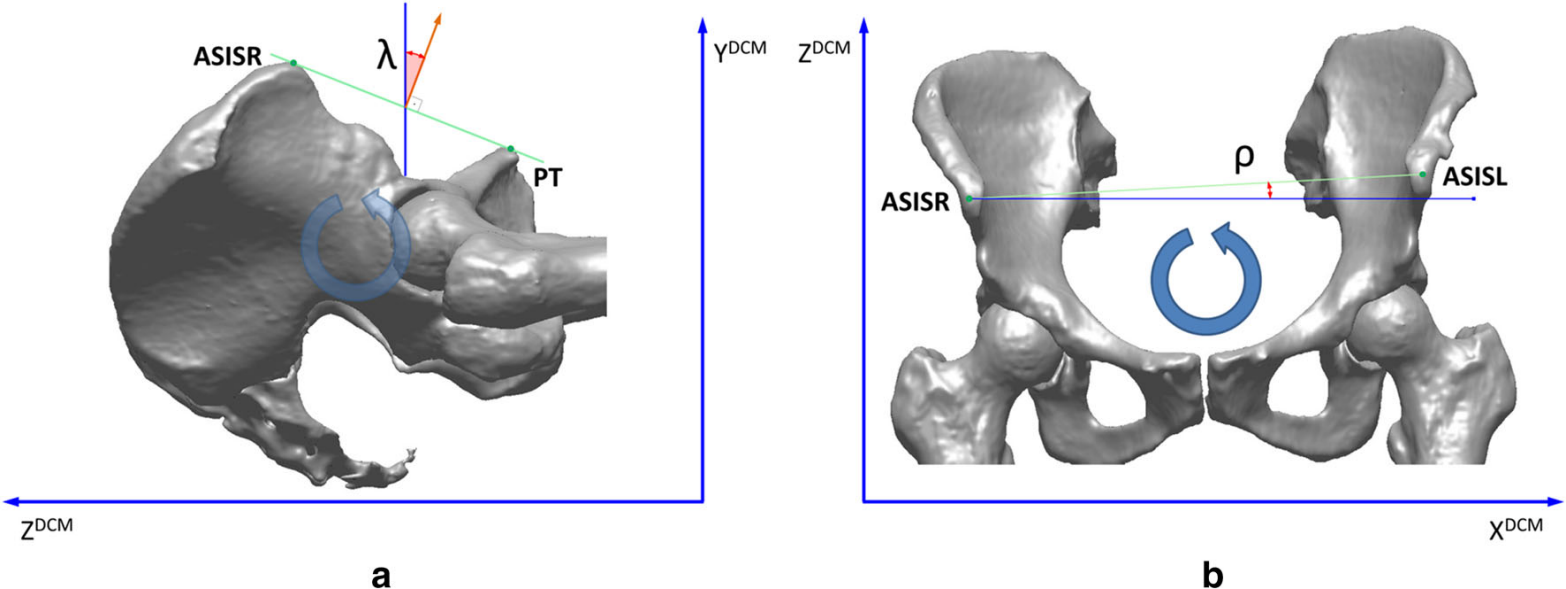
Anterior–posterior pelvic tilt estimated by angle *λ* (**a**), and lateral pelvic tilt estimated by angle *ρ* (**b**). The *X*^*DCM*^*, Y*^*DCM*^*, Z*^*DCM*^ are the principal axes of the CT scanner table, well-defined in the DICOM format of the acquired CT datasets (*X*^*DCM*^ i transverse table axis, *Y*^*DCM*^ sagittal table axis, and *Z*^*DCM*^ longitudinal table axis)

We analyzed the positioning of the patients’ pelvis during the CT by the calculation of two angles: λ and ρ. Both angles were estimated by measuring the pelvis positioning in relation to the principal axes of the CT table, defined by the DICOM coordinate system in the CT datasets. Angle *λ* reflects the rotation of the pelvis on the sagittal plane, and angle ρ reflects the rotation of pelvis in the coronal plane, as shown in Fig. 4a and b. Angle λ is defined as the angle between the normal vector of the anterior pelvis plane (coronal) and the vertical axis *Y*^*DCM*^ of the CT table. Angle ρ is defined as the angle between the vector defined by the landmarks anterior superior iliac spine right and left (ASISR and ASISL) and the transverse table axis *X*^*DCM*^.

### Single linear regression analysis of the angle λ and the difference of angles AV^3D^and AV^2D^(Δ^3D−2D^)

**Fig. 5:**
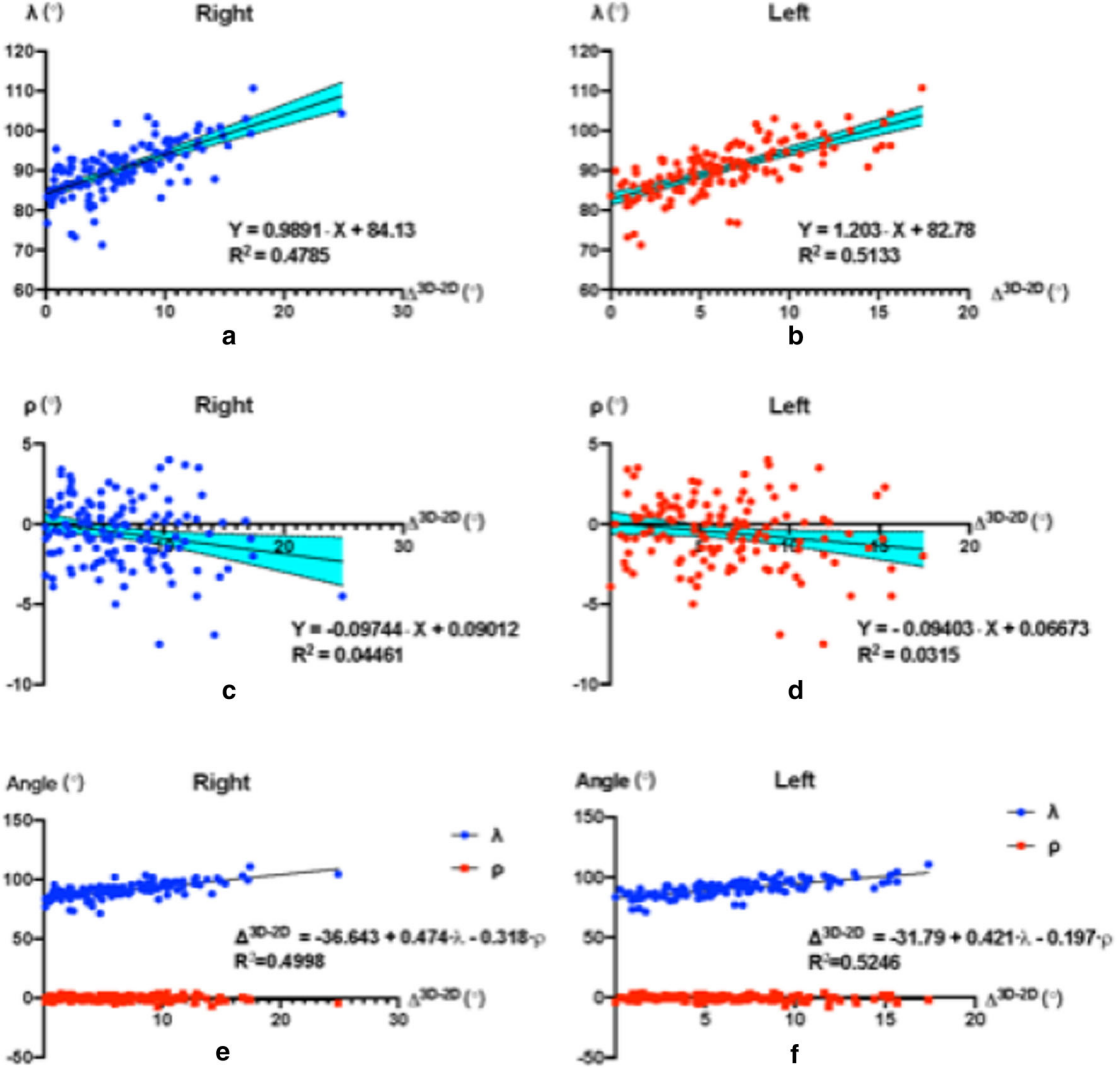
**a**, **b** On both sides (left and right), angle *λ* shows a linear regression relationship with the difference of AV angles Δ^3D−2D^. **c**,** d** On both sides, angle *ρ* showed a linear regression relationship with the difference of AV angles Δ^3D−2D^. **e**,**f** Angle *λ* and angle *ρ* have a linear regression relationship with the difference of AV angles Δ^3D−2D^ (*p* < 0.0001, *R*^2^ = 0.5246) on both the right and left side

On the right, angle λ showed a linear regression relationship with the difference of AV angles Δ^3D−2D^ (Equation: Y = 0.9891·X + 84.13, p < 0.0001, R^2^ = 0.4785, Fig. 5a). On the left, angle λ showed a linear regression relationship with the difference of AV angles Δ^3D−2D^ (Equation: Y = 1.203·X + 82.78, p < 0.0001, R^2^ = 0.5133; Fig. 5b).

### Single linear regression analysis of the angle ρ and the Δ^3D−2D^

On the right, angle ρ showed a linear regression relationship with the difference of AV angles Δ^3D−2D^ (Equation: Y = 0.09744∙X + 0.09012, *p* < 0.0001, *R*^2^ = 0.0446, Fig. 5c). On the left, angle ρ showed a linear regression relationship with the difference of AV angles Δ^3D−2D^ (Equation: Y = 0.09403∙X + 0.06673, *p* < 0.0001, *R*^2^ = 0.0315; Fig. 5d).

### Multiple linear regression analysis of the angles λ and ρ, and the Δ^3D−2D^ on the right

Angle λ and angle ρ showed a linear regression relationship with the difference of AV angles Δ^3D−2D^ on the right (*p* < 0.0001, *R*^2^ = 0.4998).

$$ \Delta^{{{\text{3D}} - {\text{2D}}}} = \, - {36}.{643 } + \, 0.{474} \cdot \lambda \, - \, 0.{318} \cdot \rho $$

The regression coefficient value of*λ* is 0.474 (t = 10.709, *p* < 0.0001), angle *λ* has a significant positive influence on* Δ*^3D−2D^ on the right side. The regression coefficient value of *ρ* is − 0.318 (t = − 2.321, *p* = 0.022 < 0.05), which means that angle ρ*ρ* has a significant negative influence on*Δ*^3D−2D^ on the right (Fig. 5e).

When considering the mutual influence between angle *λ* and angle *ρ*.

$$ \begin{aligned}  \Delta ^{{{\text{3D}} - {\text{2D}}}} = & - 34.84 + 0.4533 \cdot \lambda + 5.64 \cdot \rho - 0.06555 \cdot \lambda \cdot \rho \\  {\text{R}}^{2} =&\, 0.5401 \\ \end{aligned} $$

The regression coefficient value of λ is 0.4533 (t = 10.53, *p* < 0.0001), angle *λ* has a significant positive influence on Δ^3D−2D^ on the right. The regression coefficient value of ρ is 5.64 (t = 3.121, *p* = 0.0022 < 0.05), therefore angle *ρ* has a significant positive influence on Δ^3D−2D^ on the right. The regression coefficient value of *λ*·*ρ* is − 0.06555 (t = 3.306, *p* = 0.0012 < 0.05), therefore angle *λ*·*ρ* has a significant positive influence on Δ^3D−2D^ on the right.

### Multiple linear regression analysis of the angles λ and ρ, and the Δ^3D−2D^on the left

Angle λ and angle ρ have a linear regression relationship with the difference of AV angles Δ^3D−2D^ (*p* < 0.0001, R^2^ = 0.5246) on the left.

$$ \Delta^{{{\text{3D}} - {\text{2D}}}} = - {31}.{79} + 0.{421} \cdot \lambda - 0.{197} \cdot \rho $$

The regression coefficient value of *λ* is 0.421 (t = 11.448, p < 0.0001), angle λ has a significant positive influence on Δ^3D−2D^ on the left. The regression coefficient value of *ρ* is − 0.197 (t = − 1.728, *p* = 0.0865 > 0.05), therefore angle ρ has no significant influence on Δ^3D−2D^ on the left (Fig. 5f).

Overall, the difference between AV^3D^ and AV^2D^ was mainly caused by angle *λ* (the anterior–posterior pelvic tilt).

## Discussion

With 3D, we measured the true AV angle as 16.1° with a SD of 5.9°. The AV angle in male and female individuals was significantly different (*p* < 0.0001), both measured with the 2D and the 3D method, which can be explained by the known anatomical difference of the pelvis in men and women. We present the true range of the acetabular AV angle, which is similar to the data published before (summarized in Table 5) [15–19, 23].

**Table 5:** Different acetabular angles measured in previous studies

Ref. no.	Year	Method	Gender	n*	Criteria	AV angle [°]	SD	Range	Comments
[17]	1983	CT	Overall	86		17	6		Left/right not described
[11]	1989	CT	Overall	40	Left	19.8	5.7	Jul-30	
					Right	19	4.7	Oct-28	
			Male	23	Left	18.5	5.6	Jul-30	
					Right	18.4	4.5	Oct-25	
			Female	17	Left	21.6	5.4	Oct-30	
					Right	19.8	4.9	Nov-28	
[19]	1996	CT	Overall	60		15.7			Left/right, Male/female not analysed
[20]	2006	CT	Overall	100	Age	23	5	Dec-39	Divided by age, left/right not divided
			Male	17	< 70y	22	6	Dec-39	
				25	> 70y	22	6	13–35	
			Female	40	< 70y	23	5	15–35	
				18	> 70y	25	5	17–34	
[12]	2007	X-ray, anatomic	Overall	43	Anatomic	20.1	6.4		Left/right not analysed, male/female not analysed; comparison of anatomic and radiographic (X-ray) measurements
					Radiographic	20.3	6.5		
			Male	30					
			Female	13					
[5]	2008	3D-CT	Overall	27	Normal	17	8	Jan-31	Left/right difference not included, difference between normal and dysplastic hips
					Dysplastic	19	9	− 46	
			Male	11	Normal	15	7	Jan-24	
					Dysplastic	18	3	Dec-21	
			Female	16	Normal	18	8	Feb-31	
					Dysplastic	19	10	Jul-39	
[13]	2010	3D-CT	Overall	25	Left	17.29	5.8		Male/female differences not calculated
					Right	17.55	5.6		
			Male	11					
			Female	14					
[16]	2011	3D-CT	Overall	50	Level 1	14.4	10.5	− 53.4	Acetabular anteversion measured on different levels on the 3D model
					Level 2	21.2	8.1	− 43.3	
					Level 3	22.5	6.1	1.1–38.8	
					Level 4	21.3	5.5	8.3–34.6	
					Level 5	22.1	6.6	1.38–39.1	
			Male	25	Level 1	11.6	9.4	− 42	
					Level 2	18.2	7.4	− 30.97	
					Level 3	20	4.8	1.1–27.5	
					Level 4	18.9	5	0.7–30.47	
					Level 5	19.7	5.6	1.38–32.09	
			Female	25	Level 1	17	10.9	− 44.84	
					Level 2	24.3	7.8	5.5–40.9	
					Level 3	25.1	6.2	7.5–38.8	
					Level 4	23.6	5.5	8.3–34.6	
					Level 5	24.5	6.7	9.2–39.1	
[4]	2013	3D-CT	Overall	49	Prone	24	5.3	22.9–25.1	Difference made in between prone position and reformatted images
					Reformatted	21.3	5	20.3–22.3	
			Male	26	Prone	23.1	4.8	21.8–24.4	
					Reformatted	19.4	4.4	18.2–20.6	
			Female	23	Prone	25.1	5.6	23.4–26.8	
					Reformatted	22.8	5.3	21.2–24.4	
[10]	2014	3D-CT	Overall	200	Anatomic	23.2	6.6		Three different methods to measure acetabular anteversion
					Radiographic	19.2	5.6		
					Operative	30.6	8.6		
			Male	112	Anatomic	21.5	6.1		
					Radiographic	17.5	5		
					Operative	28	7.6		
			Female	88	Anatomic	24.7	6.6		
					Radiographic	20.5	5.8		
					Operative	32.6	8.8		
[24]	2017	3D-CT	Overall	49	Anatomic	18.12	7.59		Three different methods to measure acetabular anteversion
					Radiographic	14.3	5.64		
					Operative	24.97	9.68		
			Male	28	Anatomic	17.51	7.98		
					Radiographic	13.73	5.93		
					Operative	23.25	9.53		
			Female	21	Anatomic	18.93	7.04		
					Radiographic	15.06	5.21		
					Operative	27.25	9.51		
[25]	2017	3D-CT	Overall	100	Anatomic	20.1		5.9–33.1	
					Radiographic	16.1		4.5–26.8	
					Operative	24.9		7.0–39.2	
			Male	50	Anatomic	18.8		9.1–31.0	
					Radiographic	14.8		7.3–25.0	
					Operative	22.9		10.9–36.5	
			Female	50	Anatomic	21.5		5.9–33.1	
					Radiographic	17.3		4.5–26.8	
					Operative	26.9		7–39.2	

We compared 3D as well as 2D approaches for the same collection of CT datasets to show the true acetabular angle with the largest dataset published to our knowledge. The method is independent of the patients’ position in the CT as well as of the position of the pelvis within the body.

Our data support the already established normal values for the AV angle. Higgins et al. [10] have reported similar results after measuring the 3D angle of the acetabulum. In that study, however, the direct comparison with the most commonly used 2D method is missing. They are proposing an image processing technique for their measurements that is supposed to be automatic; however, the first three points still have to be set manually. Nevertheless, this study as well as our study helps understanding the complex anatomy of the hip.

Zhang et al. [25] found a smaller acetabular anteversion, which might be due to the fact that their patient population was mainly Asian. Therefore, our study provides the first representative collective for the European population. They also found that female acetabula were significantly more anteverted, which is consistent with our results.

Wang et al. [24] have compared the virtual measurement of the AV angle with the measurement on a printed model and have noted no difference. This is not surprising, as the model is created from computer data and therefore cannot be different from the virtually measured parameters. They also found 2D measurements not as accurate as 3D measurements, so our data matches their work.

Arora et al. [2] created the Perth hip protocol measuring the acetabular anteversion in a cadaver and comparing it to a virtual 3D model. They also concluded that 3D measurements are more precise than 2D measurements.

CT scans expose patients to radiation and recent studies show that MRI scans can provide very similar results [8]. This is promising, especially for pediatric cases. Neurologically impaired and/or very young patients who might require anesthesia for an MRI are yet problematic.

2D measurements can lead to false assumptions of the AV angle. The angle is over-estimated, and only slightly retroverted acetabula might be evaluated as normal. This is dangerous especially in dysplastic hips, since it could lead to false clinical decision making and be a pitfall in planning pelvic osteotomies. It would be interesting to investigate if the measurements acquired with the 2D method causes higher surgery rates for example in anteverting pelvic osteotomies and revision surgery rates. Intraoperative imaging is still done with a C-arm, which only allows 2D assessment on fluoroscopy. We therefore see a role for 3D navigated surgery to aid in pelvic osteotomies [3, 6, 26]. Another promising approach is augmented reality in surgery [1] to assist with a better 3D understanding. To date, these options are under investigation, but not established for clinical use yet. We would encourage further research in that field.

Since the pelvic tilt is not considered in the 2D method, it has an obvious disadvantage in comparison to the 3D method, where the AV angle estimation cannot be biased by congenital pelvic tilt or inappropriate patient positioning on the CT table (see Fig. 3). Our statistical analyses revealed a substantial discrepancy between the AV angles estimated with the 3D method and the AV angles estimated with the 2D method, which was mainly caused by the anterior–posterior pelvic tilt. Therefore, we believe that the 3D method supports the surgeon better in determining the true acetabular angle. This could be useful for the placement of total hip arthroplasty, and also for its revision surgery. 3D CT scans are already used to plan multiple procedures such as revision of hip arthroplasties or osteotomies [26].

After training, it took our examiners about 30 to 45 min to calculate the true acetabular AV angle, depending on the quality of the data and the configuration of the anatomy. A crucial factor in this process is the segmentation of the bony structures in the CT data. The used software might not be ready for routine use yet, but it provides a true anatomical parameter and helps understanding the anatomy of the acetabulum and therefore the hip joint. Abnormal morphology of the acetabular rim, for example, caused by bony hyperplasia due to osteophytes, pincer deformity or cartilage calcification, may cause an incorrect delineation of the acetabular ridge at the segmentation stage. As a result, the estimation of the acetabular plane (and thus the acetabular angle) will be biased. This is one of the limitations of our study.

The 3D method is reliable and safe. Our mathematical description of the pelvis positioning shows the factors responsible for the deviation between 2 and 3D AV angle. This quantitative examination of systematic bias distinguishes our work from the other studies listed in Table 5.

## Conclusion

3D measurement should be the gold standard for measuring the AV angle. There is a significant difference in the evaluation of the AV angle by 3D and 2D. The difference is mainly caused by the anterior–posterior pelvic tilt. The main disadvantage of the 3D method is the time-consuming CT data segmentation.

Being aware of the normal anatomy of the acetabulum is essential for the diagnosis and treatment of acetabular deformities.

The true 3D AV angle will help improving operation techniques and provide the best possible care for patients in the future.
