# Correction to: The “true” acetabular anteversion angle (AV angle): 2D CT versus 3D model

**DOI:** 10.1007/s11548-022-02734-9

**Published:** 2022-10-06

**Authors:** Kira A. Barlow, Zdzislaw Krol, Pawel Skadlubowicz, Chao Dong, Vanja Zivkovic, Andreas H. Krieg

**Affiliations:** 1 Pediatric Orthopedic Department, University Children’s Hospital Basel (UKBB), Spitalstrasse 33, 4056 Basel, Switzerland; 2 Institute of Computer Science, Department of Biomedical Computer Systems, Faculty of Computer and Materials Science, University of Silesia, Sosnowiec, Poland

## Correction to: International Journal of Computer Assisted Radiology and Surgery 10.1007/s11548-022-02717-w

The original version of this article unfortunately contained a mistake. The wrong Table 2 was published and in Table 5, document measures in the column “Range” were mistakenly listed as dates.

The corrected Tables 2 and 5 is given in the following page.

**Table 2:** Comparison between AV^3D^ and AV^2D^ angle estimation methods, over all patients, in males and females, and in the right and left subgroups

	Overall	Male	Female	*p*-value*	Right	Left	*p*-value***
	*n* = 258	*n* = 136	*n* = 122		*n* = 129	*n* = 129	
AV^3D^, m (SD) (Range)	16.1 (5.9) (0.2–31.2)	14.0 (5.4) (0.2–28.8)	18.4 (5.6) (3.0–31.2)	< 0.0001	16.4 (5.8) (0.89–30.9)	15.8 (5.10) (0.2–31.2)	< 0.0001
AV^2D^, m (SD) (Range)	22.0 (6.0) (5.0–40.1)	20.3 (4.9) (9.2–33.6)	23.9 (6.5) (5.0–40.1)	< 0.0001	22.3 (6.0) (6.8–39.8)	21.7 (8.9) (5.0–40.1)	< 0.0001
Difference between mean (2D-3D), m (SD)	5.8 (4.9)	6.2 (4.5)	5.5 (5.4)		5.9 (5.2)	5.8 (4.7)	
95% Confidence Interval (CI)	5.3–6.5	5.5–7.0	4.6–6.5		5.0–6.8	5.0–6.7	
**p-value	< 0.0001	< 0.0001	< 0.0001		< 0.0001	< 0.0001	

*Comparison between male and female, **Comparison between 3 and 2D method, ***Comparison between left and right side

**Table 5:** Different acetabular angles measured in previous studies

Ref. Nr.	Year	Method	Gender	n*	Criteria	AV Angle (°)	SD	Range	Comments
17	1983	CT	Overall	86		17	6		Left/right not described
11	1989	CT	Overall	40	Left	19.8	5.7	7–30	
					Right	19.0	4.7	10–28	
			Male	23	Left	18.5	5.6	7–30	
					Right	18.4	4.5	10–25	
			Female	17	Left	21.6	5.4	10–30	
					Right	19.8	4.9	11–28	
19	1996	CT	Overall	60		15.7			Left/right, Male/female not analysed
20	2006	CT	Overall	100	Age	23	5	12–39	Divided by age, left/right not divided
			Male	17	< 70y	22	6	12–39	
				25	> 70y	22	6	13–35	
			Female	40	< 70y	23	5	15–35	
				18	> 70y	25	5	17–34	
12	2007	X-ray, anatomic	Overall	43	Anatomic	20.1	6.4		Left/right not analysed, male/female not analysed; comparison of anatomic and radiographic (X-ray) measurements
					Radiographic	20.3	6.5		
			Male	30					
			Female	13					
5	2008	3D-CT	Overall	27	Normal	17	8	1–31	Left/right difference not included, difference between normal and dysplastic hips
					Dysplastic	19	9	− 7–39	
			Male	11	Normal	15	7	1–24	
					Dysplastic	18	3	12–21	
			Female	16	Normal	18	8	2–31	
					Dysplastic	19	10	7–39	
13	2010	3D-CT	Overall	25	Left	17.29	5.8		Male/female differences not calculated
					Right	17.55	5.6		
			Male	11					
			Female	14					
16	2011	3D-CT	Overall	50	Level 1	14.4	10.5	− 12.9–40.5	Acetabular anteversion measured on different levels on the 3D model
					Level 2	21.2	8.1	− 2.4–40.9	
					Level 3	22.5	6.1	1.1–38.8	
					Level 4	21.3	5.5	8.3–34.6	
					Level 5	22.1	6.6	1.38–39.1	
			Male	25	Level 1	11.6	9.4	− 12.9–29.1	
					Level 2	18.2	7.4	− 2.4–28.57	
					Level 3	20.0	4.8	1.1–27.5	
					Level 4	18.9	5	0.7–30.47	
					Level 5	19.7	5.6	1.38–32.09	
			Female	25	Level 1	17.0	10.9	− 4.34–40.5	
					Level 2	24.3	7.8	5.5–40.9	
					Level 3	25.1	6.2	7.5–38.8	
					Level 4	23.6	5.5	8.3–34.6	
					Level 5	24.5	6.7	9.2–39.1	
4	2013	3D-CT	Overall	49	Prone	24	5.3	22.9–25.1	Difference made in between prone position and reformatted images
					Reformatted	21.3	5.0	20.3–22.3	
			Male	26	Prone	23.1	4.8	21.8–24.4	
					Reformatted	19.4	4.4	18.2–20.6	
			Female	23	Prone	25.1	5.6	23.4–26.8	
					Reformatted	22.8	5.3	21.2–24.4	
10	2014	3D-CT	Overall	200	Anatomic	23.2	6.6		Three different methods to measure acetabular anteversion
					Radiographic	19.2	5.6		
					Operative	30.6	8.6		
			Male	112	Anatomic	21.5	6.1		
					Radiographic	17.5	5.0		
					Operative	28.0	7.6		
			Female	88	Anatomic	24.7	6.6		
					Radiographic	20.5	5.8		
					Operative	32.6	8.8		
24	2017	3D-CT	Overall	49	Anatomic	18.12	7.59		Three different methods to measure acetabular anteversion
					Radiographic	14.30	5.64		
					Operative	24.97	9.68		
			Male	28	Anatomic	17.51	7.98		
					Radiographic	13.73	5.93		
					Operative	23.25	9.53		
			Female	21	Anatomic	18.93	7.04		
					Radiographic	15.06	5.21		
					Operative	27.25	9.51		
25	2017	3D-CT	Overall	100	Anatomic	20.1		5.9–33.1	
					Radiographic	16.1		4.5–26.8	
					Operative	24.9		7.0–39.2	
			Male	50	Anatomic	18.8		9.1–31.0	
					Radiographic	14.8		7.3–25.0	
					Operative	22.9		10.9–36.5	
			Female	50	Anatomic	21.5		5.9–33.1	
					Radiographic	17.3		4.5–26.8	
					Operative	26.9		7–39.2	

In the section “Single linear regression analysis of the angle ρ and the Δ^3D−2D”^

Both equations should have a “minus” sign in the beginning (as in Figure 5c and 5d):

(Equation: Y = 0.09744∙X + 0.09012, *p* < 0.0001, *R*^2^ = 0.0446, Fig. 5c). On the left, angle ρ showed a linear regression relationship with the difference of AV angles Δ^3D−2D^ (Equation: Y = 0.09403∙X + 0.06673, *p* < 0.0001, *R*^2^ = 0.0315; Fig. 5d).

It should be:

(Equation: Y = − 0.09744∙X + 0.09012, *p* < 0.0001, *R*^2^ = 0.0446, Fig. 5c). On the left, angle ρ showed a linear regression relationship with the difference of AV angles Δ^3D−2D^ (Equation: Y = − 0.09403∙X + 0.06673, *p* < 0.0001, *R*^2^ = 0.0315; Fig. 5d).

In the section “Multiple linear regression analysis of the angles λ and ρ, and the Δ^3D−2D^ on the right" Rho-angle was mentioned double:

“which means that angle ρ*ρ* has a significant negative influence on *Δ*^3D−2D^ on the right (Fig. 5e)”

It should be:

“which means that angle ρ has a significant negative influence on *Δ*^3D−2D^ on the right (Fig. 5e)”.

